# Future Path Toward TB Vaccine Development: Boosting BCG or Re-educating by a New Subunit Vaccine

**DOI:** 10.3389/fimmu.2018.02371

**Published:** 2018-10-16

**Authors:** Nancy Gupta, Saurabh Garg, Satish Vedi, Dennis Y. Kunimoto, Rakesh Kumar, Babita Agrawal

**Affiliations:** ^1^Department of Laboratory Medicine and Pathology, Faculty of Medicine and Dentistry, University of Alberta, Edmonton, AB, Canada; ^2^Department of Medicine, Faculty of Medicine and Dentistry, University of Alberta, Edmonton, AB, Canada; ^3^Department of Surgery, Faculty of Medicine and Dentistry, University of Alberta, Edmonton, AB, Canada

**Keywords:** vaccine, tuberculosis, subunit, BCG, booster, cellular immunity

## Abstract

Tuberculosis (TB), an infectious disease caused by *Mycobacterium tuberculosis* (*Mtb*), kills 5,000 people per day globally. Rapid development and spread of various multi drug-resistant strains of *Mtb* emphasize that an effective vaccine is still the most cost-effectives and efficient way of controlling and eradicating TB. Bacillus Calmette-Guerin (BCG), the only licensed TB vaccine, still remains the most widely administered human vaccine, but is inefficient in protecting from pulmonary TB in adults. The protective immunity afforded by BCG is thought to wane with time and considered to last only through adolescent years. Heterologous boosting of BCG-primed immune responses using a subunit vaccine represents a promising vaccination approach to promote strong cellular responses against *Mtb*. In our earlier studies, we discovered lipopeptides of ESAT-6 antigen with strong potential as a subunit vaccine candidate. Here, we have investigated that potential as a booster to BCG vaccine in both a pre-exposure preventive vaccine and a post-exposure therapeutic vaccine setting. Surprisingly, our results demonstrated that boosting BCG with subunit vaccine shortly before *Mtb* challenge did not improve the BCG-primed immunity, whereas the subunit vaccine boost after *Mtb* challenge markedly improved the quantity and quality of effector T cell responses and significantly reduced *Mtb* load in lungs, liver and spleen in mice. These studies suggest that ESAT-6 lipopeptide-based subunit vaccine was ineffective in overcoming the apparent immunomodulation induced by BCG vaccine in *Mtb* uninfected mice, but upon infection, the subunit vaccine is effective in re-educating the protective immunity against *Mtb* infection. These important results have significant implications in the design and investigation of effective vaccine strategies and immunotherapeutic approaches for individuals who have been pre-immunized with BCG vaccine but still get infected with *Mtb*.

## Introduction

Tuberculosis (TB), caused by infection with *Mycobacterium tuberculosis* (*Mtb*), is the leading cause of death due to an infectious disease, claiming ~2 million lives globally each year (1). The rise in strains of *Mtb* resistant to almost all of the available TB drugs is making this deadly infectious disease ominous. Moreover, one third of the world's population harbors *Mtb* in a latent state serving as a large reservoir. These individuals are potentially at risk of developing active disease again and further spreading it easily to the remaining population (2, 3). Currently, Bacille Calmette-Guerin (BCG), which was developed nearly 100 years ago, is the only available vaccine to prevent TB. BCG remains the most widely administered vaccine around the world and is effective in providing 60–80% protection against childhood and extra-pulmonary forms of TB (4–7). But the beneficial effect of the vaccine given to children wanes over time. Further, BCG affords highly inconsistent and inadequate protection against pulmonary TB and is ineffective when given in adulthood. Because BCG has minimal effect on pulmonary TB, it has not had major effect on the global burden of TB (4–7). The reasons for the varying protective efficacy of BCG are still unclear. It is thought that pre-exposure with environment mycobacteria may prevent or mask the protective immunity induced by BCG. Also, BCG strain(s) (have) lost some of the genes encoding immunodominant antigens during attenuation (8). However, considering the fact that BCG has been administered to >3 billion infants in countries/settings with a high incidence of TB, and this will continue in the foreseeable future, developing a new and more efficient vaccine to replace BCG is a formidable task (9). Therefore, improving, boosting or supplementing BCG in different clinical settings appears to be a more logical path for new vaccine/immunotherapy approaches for TB.

In attempts to improve the efficacy of BCG vaccine, a phase 1 clinical trial for safety and tolerability was conducted using a recombinant BCG that expressed a) immunodominant antigens seen in active infection and during reactivation from latency (Ag85A, Ag85B and Rv3407) and b) a mutant perfringolysin (PFOG137Q) derived from *Clostridium perfringens*, able to perforate the endosomal membrane (10, 11). However, for some unknown reasons, 2 of 8 patients developed reactivation of varicella zoster virus, resulting in the discontinuation of further development of this vaccine (11).

Heterologous prime-boost strategies using viral-vectored vaccines or adjuvanted protein/peptide subunit vaccines have been a hopeful approach to TB vaccination. Several viral vectors, such as adenovirus (human Ad5, human Ad35, Chimp ChAdOx1), vesicular stomatitis virus (VSV), modified vaccinia Ankara (MVA) expressing antigen 85A, a highly conserved molecule across mycobacterial species, have been shown to provide superior protection when administered in BCG-primed animals compared to BCG alone (12–17). However, despite being highly immunogenic, none of these experimental vaccines provided satisfactory results in clinical testing yet (18).

Recombinant fusion protein-based vaccines may have potential as a booster to BCG vaccine and may also possess a number of advantages such as high level of safety, purity and cost-effectiveness. However, these vaccines would require effective and safe adjuvant to strengthen the immune responses generated. Some of the fusion protein-based vaccines being tested in clinical trials include H56:IC31 [a fusion protein of three *M. tuberculosis* antigens (85B, ESAT-6 and Rv2660c) formulated in the proprietary adjuvant IC31® from Valneva], H4:IC31 [a recombinant fusion protein of *Mtb* antigens 85B and TB10.4 combined with IC31® adjuvant] and M72 + ASO1E [immunogenic fusion protein (M72) derived from two *M. tuberculosis* antigens (MTB32A and MTB39A), and the GlaxoSmithKline's proprietary adjuvant AS01E] (19–24). The efficacy of these experimental vaccines remains to be determined.

Early secreted antigenic target 6 kDa protein (ESAT-6) is a potent T-cell antigen expressed in pathogenic *Mtb* and contains an unusually high number of permissive T cell epitopes spanning the entire sequence of the molecule (25, 26). ESAT-6 is an interesting antigen associated with active *Mtb* infection; however, the gene encoding ESAT-6 belongs to the RD-1 region, and all BCG vaccine strains distributed worldwide have deleted RD1 regions (27). ESAT-6 based subunit vaccines have shown tremendous potential in animal models (28, 29).

We have earlier reported the lipopeptides of ESAT-6 antigen corresponding to immunodominant epitopes as a promising candidate for TB vaccine. We demonstrated that mucosal immunization with lipopeptides of ESAT-6 antigen with or without adjuvant monophosphoryl lipid A (MPL) promoted strong mucosal and systemic immune responses, which were effective in reducing the *Mtb* burden in a mouse model of *Mtb* infection, supporting its efficacy as a prophylactic vaccine (30).

In the present study, we investigated the potential of lipopeptides of ESAT-6 based subunit vaccine to boost protective immunity induced by BCG both before (pre-exposure) and after (post-exposure) infection. We hypothesized that lipopeptides of ESAT-6 based subunit (LP-ESAT-6) vaccine could be potentially used to boost BCG as a prophylactic vaccine and used as immunotherapeutic vaccine for BCG vaccinated but still infected individuals. Intriguingly, while the pre-exposure boost with our LP-ESAT-6 vaccine did not lead to significant improvement in efficacy of BCG upon infection of mice with *Mtb*, post-exposure LP-ESAT-6 boost in BCG primed mice led to a significant decrease in *Mtb* bacterial loads compared to the BCG vaccine group, which was associated with increased immune responses both locally in lungs and systemically in spleen. While these results further expose the difficulties encountered in designing effective strategies to boost BCG vaccine, they provide a new paradigm to the concept of boosting BCG vaccine in efforts to investigate novel approaches for TB vaccine.

## Materials and methods

### Mice

All animal experiments used in this study were approved by the University of Alberta's Animal Care and Use Committee (ACUC) for Health Sciences and conducted in accordance with the guidelines of the Canadian Council of Animal Care (CCAC). Five-to six-week-old female BALB/c mice were purchased from Charles River Laboratories and housed in biocontainment BSL 2/3 animal facility (HSLAS) at the University of Alberta.

### Synthetic peptides, antigen and adjuvants

Synthetic lipopeptides derived from ESAT-6 [P1 (ESAT-6_1−15_): MTEQQWNFAGIEAAAK(palmitate)G; P4 (ESAT-6_31−55_):EGKQSLTKLAAAWGGSGSEAYQGVQK(palmitate)G; P5 (ESAT-6_46−70_): SGSEAYQGVQQKWDATATELNNALQK(palmitate)G; P6 (ESAT-6_61−85_):TATELNNALQNLARTISEAGQAMASK(palmitate)G; P7 (ESAT-6_76−95_):ISEAGQAMASTEGNVTGMFAK(palmitate)G], were custom synthesized by Genscript Inc. (NJ, United States) (30). All lipopeptides were dissolved in DMSO at 10 mg/ml, stored at −20°C, and diluted with PBS or medium prior to use. MPL (Sigma Aldrich) was used as adjuvant. PPD antigen was obtained from Statens Serum Institut (Denmark).

### *Mycobacterium bovis* BCG and *Mtb* H37Ra strains

BCG vaccine (Copenhagen) and *M. tuberculosis* (H37Ra) (ATCC, Rockville, MD) were grown in Middlebrook 7H9 broth supplemented with 10% oleic acid albumin dextrose complex (BD), 0.05% Tween 80, and 0.5% glycerol to mid-log phase before freezing at −80°C. Bacterial viability was determined by plating samples on Middlebrook 7H11 medium supplemented with ODAC and counting the number of colony-forming units (CFUs).

### BCG vaccination and immunization schedule

**Figure 1:**
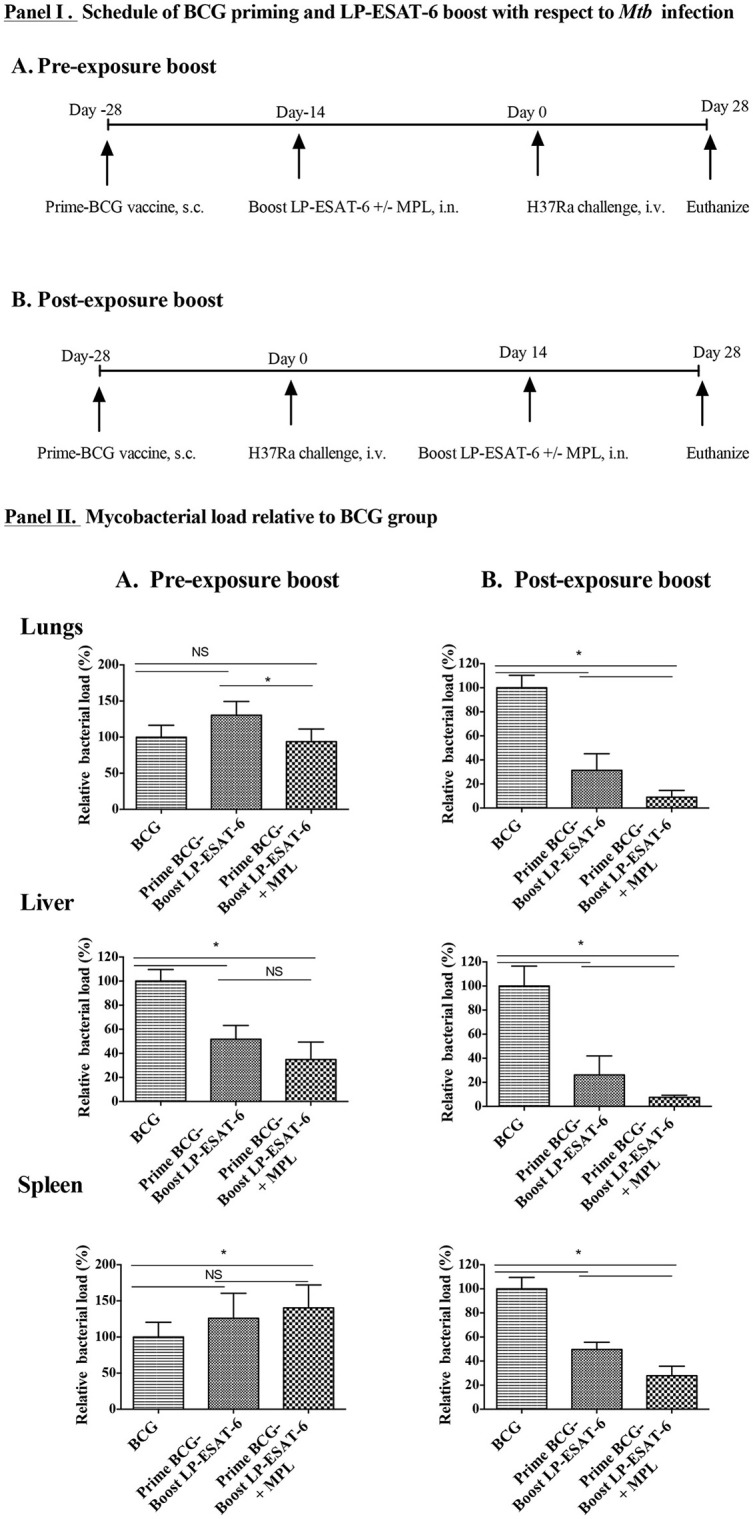
Decrease in *Mtb* loads is dependent on schedule of the boost in heterologous prime-boost with BCG/LP-ESAT-6. **(I)**. Experimental design of BCG priming (s.c.) and LP-ESAT-6 boost (i.n.) using pre- and post-exposure schedules. **(II)**. Female BALB/c mice (*n* = 5/group) were immunized subcutaneously with BCG (1 x 10^6^ live BCG, 100 μl/mouse) or PBS (unvaccinated group). A mixture of P1 and P4-P7 lipopeptides (12 μg each) alone and combined with MPL was administered intranasally as LP-ESAT-6 boost according to the schedule shown in **(I)**, either before or after infection with H37Ra (0.5 × 10^6^ cfu) intravenously. Four weeks after *Mtb*-challenge, mice were euthanized and lungs, liver and spleens were collected from all groups. Bacterial loads were determined in lungs, liver and spleen by CFU assay. Mycobacterial reduction in **(A)** pre-exposure boost and **(B)** post-exposure boost of immunization is presented as percent relative bacterial load with respect to the corresponding BCG-alone group. All results are shown as mean ± standard deviation of percent relative bacterial load from individual mice. Data are representative of two different repeated experiments. ^*^Indicates significant difference (*P* < 0.05) between the compared groups and “NS” represents not significant (*P* > 0.05).

For vaccination, 1 × 10^6^ live BCG (100 μl/mouse) in PBS was administered subcutaneously (s.c.) at the base of the tail of each mouse. For subunit boost immunization, mice were administered intranasally (30 μl, 15 μl in each nostril) with a pool of lipopeptides (LP-ESAT-6, containing lipopeptide mix of P1, P4, P5, P6, and P7, each at 12 μg/mouse, total 60 μg/mouse) in the absence or presence of 10 μg of the adjuvant MPL. Control mice were immunized with an equal amount of PBS. The schedule of administration of BCG and LP-ESAT-6 are shown in Figure 1I in both pre-exposure and post-exposure settings.

### Mycobacterial challenge of mice and CFU assay

For mycobacterial infection, mice were injected with 5 × 10^5^ CFU/mouse of *Mtb* H37Ra intravenously. Four weeks after *Mtb* infection, mice were euthanized, and lungs, liver and spleen were removed aseptically and individually homogenized in 5 ml of saline. A 100 μl aliquot was taken from each organ homogenate of individual mice, serially diluted and plated on 7H11 Middlebrook agar plates (BD Biosciences). The plates were incubated at 37°C for 3–4 weeks prior to counting the colonies. CFUs of *Mtb* obtained in the unvaccinated group from individual mice in different experiments were in the ranges 5–6.5 × 10^4^ (lungs); 1.8–3.5 × 10^4^ (liver) and 0.7–1.4 × 10^4^ (spleen).

### Bronchoalveolar lavage (BAL)

To harvest BAL fluid and cells, lungs were lavaged with 500 μl of ice-cold sterile PBS [with 0.3% wt/vol bovine serum albumin (BSA)] and two 500 μl PBS washes. Fluids were centrifuged at 1,500 rpm for 10 min, and RBC lysis was performed on cell pellets. For RBC lysis, the cell pellet was resuspended in 500 μl of sterile distilled water and vortexed briefly. Immediately after, 500 μl of 2X PBS was added, the tube was vortexed briefly and the volume was made to 2 ml with 1X PBS. The obtained lymphocytes were used for staining. The supernatants of the initial 500 μl BAL fluid were used for cytokine analyses.

### Cytokine ELISA

Cytokines secreted in BAL were measured using sandwich ELISA kits (IFN-γ, IL-17A, IL-22 and IL-10) following the manufacturer's protocol (eBioscience, CA, United States). A dilution of 1:2–1:10 was used for the samples with the standards ranging from 2 to 2,000 pg/ml. The ELISA plates were read with an automated ELISA plate reader (Fluostar Optima, BMG Labtech GmbH, Ortenberg, Germany).

### T cell proliferation assay

Antigen-specific T cell proliferation assays were performed using splenocytes purified by nylon wool as reported previously by us Krishnadas et al. (31) Respective lipopeptides were used as recall antigen at a concentration of 10 μg/ml, and purified protein derivative of *Mtb* (PPD) was used at 1 μg/ml. Plates were incubated for 4 days, and cells were pulsed with 0.5 μCi/well [^3^H]-thymidine (Amersham) for 12–18 h and harvested on filter papers. The levels of [^3^H]-thymidine incorporated into the DNA of proliferating cells were counted in a Microbeta Trilux liquid scintillation counter (Perkin Elmer). Stimulation indices were calculated as CPM of antigen-stimulated culture/CPM of medium stimulated culture. Data are represented as the mean stimulation indices ± SD (standard deviation) of triplicate cultures.

### Flow cytometry analysis of immune cells

A total of 1 × 10^6^ cells in BALs from immunized and challenged mice were stained with extracellular (anti-mouse CD3e-FITC, CD4-APC, CD8-APC-Cy-7, CD69-PECy-5) (eBioscience, CA, United States) markers using established procedures (32). For intracellular cytokine staining, 2 × 10^6^ splenocytes were cultured for 4 days with medium only, peptide pools (1 μg/ml of each lipopeptide) or PPD (1 μg/ml). On day 5, brefeldin A (1.5 μg/ml, 1 X; eBioscience) was added, and cultured for 5 h at 37°C and subsequently stained for extracellular markers CD3-PE Cy7, CD4-PE Cy5, CD8-APC-Cy7, and intracellular cytokines IFN-γ-PE and IL-10-FITC using our previously reported procedures. For intracellular GrB staining, 2 x 10^6^ splenocytes obtained from mice were stained for extracellular markers CD3-PE Cy7, CD49b-Alexaflour 700, CD8-APC-Cy7, and intracellular GrB-Alexaflour 647, without *ex vivo* stimulation, using our previously reported procedures (31). Samples were run on LSR Fortessa SORP flow cytometer and analyzed using FACS-DIVA software (Becton Dickinson, Mountain View, CA, United States). Respective isotype-matched control antibodies were used to gate non-specific staining in each experiment. Gates were set to exclude 95% of isotype control antibody stained cells in all extracellular and intracellular staining experiments.

### Statistical analysis

Data were analyzed using Graphpad Prism software (Graphpad Software Inc., CA, United States). Data were presented as mean ± SD of 3-5 replicate values and significant differences between two groups or more than two groups were analyzed using two-way ANOVA using Tukey's multiple comparisons test. A *P* ≤ 0.05 was considered to be statistically significant.

## Results

### Scheduling of the intranasal boost with LP-ESAT-6 after BCG priming influences protection against *Mtb* infection

In conventional prime-boost strategies for immunization both priming and boosting vaccines are given in a prophylactic manner to prevent an infection from occurring. However, besides examining the role of LP-ESAT-6 boost to BCG vaccine in a conventional manner, we also wanted to examine if LP-ESAT-6 boost will be useful for *Mtb* infection in the settings where one had been primed with BCG vaccine and yet became infected with *Mtb*. We designed two prime-boost schedules (Figures 1IA,B) of immunizations. In schedule A, pre-exposure boost, mice were primed with BCG vaccine (s.c.), followed by LP-ESAT-6±MPL boost (intranasally) at 14 days, and infection with *Mtb* subsequently on day 14 after the boost. In schedule B, post-exposure boost, mice were primed with BCG vaccine (s.c.), followed by infection with *Mtb* on day 14, and boost with LP-ESAT-6±MPL (intranasally) subsequently 14 days after the infection. The memory responses would take 4–6 weeks to emerge, however, we wanted to examine the effect of primary immunization. A significant number of papers in immunization field use 8–14 days post-immunization to evaluate the induction of adaptive immunity. Vast literature suggests that at 14 days after immunization, both humoral and cellular adaptive immune responses can be measured. Further, the effect of adjuvant (MPL) used is on innate immunity and not expected to last for 14 days, as we had 14 days between immunization and challenge (schedule A) or euthanization (schedule B). This schedule allowed us to maintain a consistent time-frame after initial BCG priming in both pre- and post-exposure boost schedules. Mice vaccinated with BCG alone or unvaccinated mice were used as controls. Mice in both prime-boost schedules A and B were euthanized 28 days post infection with *Mtb*. The outcomes of infection were determined by examining viable counts of *Mtb* in lungs, liver and spleen, using colony forming unit (CFU) assays. Immunization with BCG-alone led to ~30–60% reduction in CFUs in lungs (~30%), liver (~60%), and spleen (~50%), compared to unvaccinated controls in both sets of experiments. CFUs of *Mtb* obtained in the unvaccinated group from individual mice in different experiments were in the ranges 5.0–6.5 × 10^4^ (lungs); 1.8–3.5 × 10^4^ (liver) and 0.7–1.4 × 10^4^ (spleen). To determine the effect of LP-ESAT-6 subunit vaccine boost over BCG priming in both schedules, we calculated % *Mtb* viable counts relative to the BCG-alone group as shown in Figures 1IIA,B. Boosting with LP-ESAT-6 subunit vaccine in pre-exposure settings did not enhance the protection over BCG-alone based on lungs and spleen but not liver (Figure 1IIA). In fact, the *Mtb* loads were slightly higher in the LP-ESAT-6 ± MPL boosted groups in both lungs and spleen compared to BCG-alone group.

Intriguingly, when BCG-primed animals were boosted with LP-ESAT-6 subunit vaccine after *Mtb* infection as in post-exposure schedule, there was a remarkable reduction in pulmonary (~30%) and extra pulmonary bacterial loads (30–50% in liver and spleen) when compared to BCG-alone vaccinated mice (Figure 1IIB). Moreover, addition of adjuvant MPL to LP-ESAT-6 subunit vaccine further enhanced the protective efficacy of booster vaccine (Figure 1IIB). Overall, these results demonstrate that the LP-ESAT-6 subunit boost with or without MPL imparts superior protection over BCG when administered after infection.

### Pre- and post-exposure boosting with LP-ESAT-6 subunit vaccine distinctly influences cytokines in bronchoalveolar lavage (BAL) fluid

**Figure 2:**
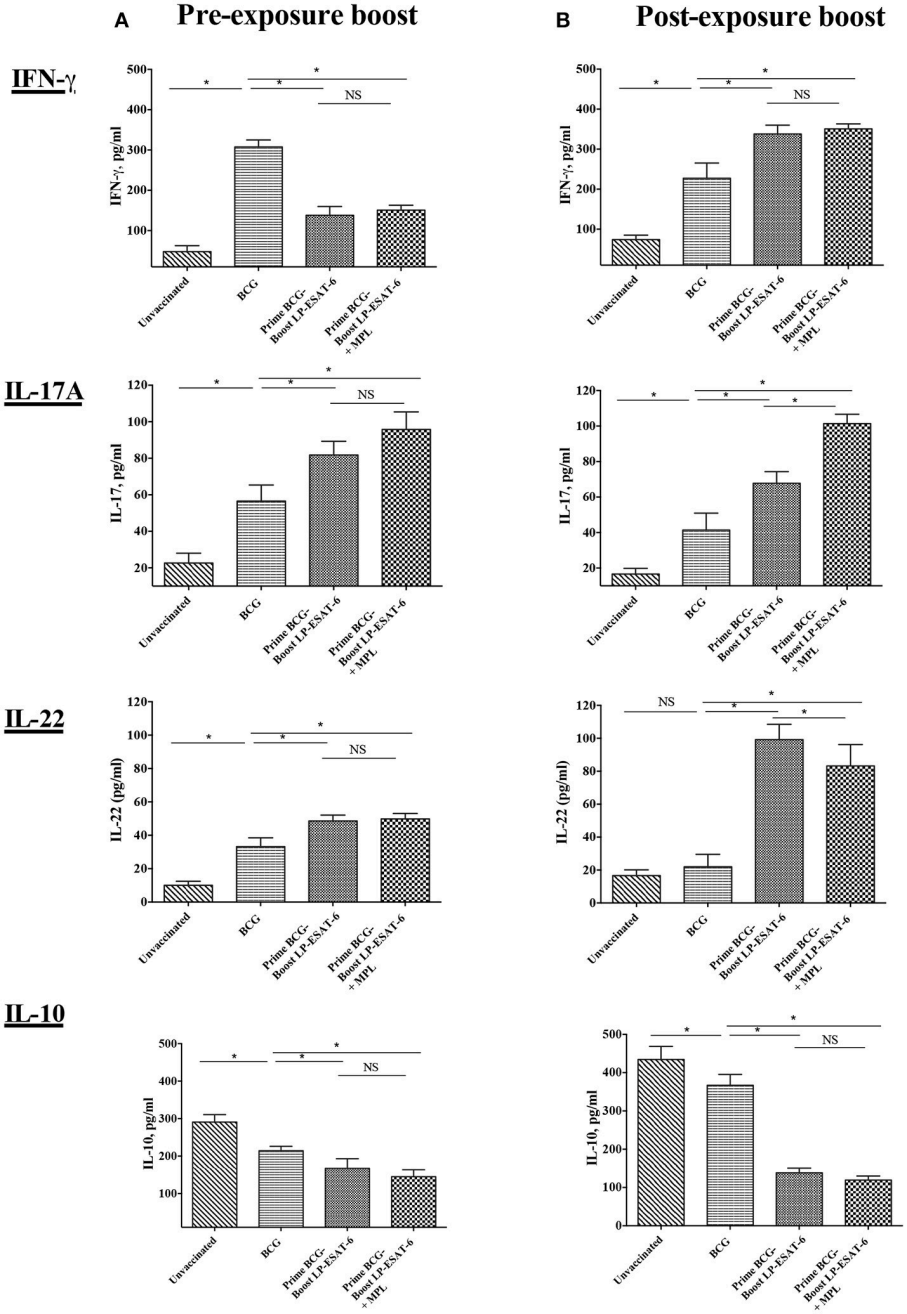
Schedule of boosting with LP-ESAT-6 subunit vaccine in BCG-primed mice leads to differential induction of cytokines in bronchoalveolar lavage fluids. Female BALB/c mice (*n* = 5/group) were immunized according to the pre-exposure or post-exposure schedule shown in Figure 1. Four weeks after infection, mice were euthanized and lung lavages were collected from **(A)** pre-exposure boost and **(B)** post-exposure boost groups to determine IFN-γ, IL-17A, IL-22 and IL-10 by ELISA. Mean ± standard deviation of cytokine concentrations from five individual mice are shown. ^*^Indicates significant difference (*P* < 0.05) between the compared groups and “NS” represents not significant (*P* > 0.05). Data are representative of two different repeated experiments.

The presence of cytokines in BAL fluid reflects the immune responses ongoing in lungs, the primary site of *Mtb* infection. To determine the pulmonary immune responses underlying the enhanced protection observed with post-exposure LP-ESAT-6 boost, we next analyzed the concentrations of IFN-γ, IL-17A, IL-22, and IL-10 in BAL fluids of vaccinated and *Mtb*-infected mice. BALs of unvaccinated animals exhibited very low levels of IFN-γ, IL-17A, and IL-22 but high levels of IL-10 (Figures 2A,B). Vaccination with BCG alone led to increased levels of IFN-γ, IL-17A, and IL-22 and a decrease in IL-10 compared to the unvaccinated group. However, interesting changes in BAL cytokines were observed upon the LP-ESAT-6 boost. Correlating to *Mtb* loads, when BCG primed mice were boosted with LP-ESAT-6 subunit vaccine after infection with *Mtb*, there were higher levels of IFN-γ, IL-17A, and IL-22 and lower levels of IL-10 compared to BCG-vaccinated mice, demonstrating the increase in protective immunity (Figure 2B). In contrast, our results showed significant decreases in the levels of IFN-γ and IL-10, and increase in IL-17A levels in the BAL fluid of BCG-primed mice boosted with LP-ESAT-6 vaccine before *Mtb* challenge, compared to BCG alone vaccinated animals (Figure 2A). No significant difference in IL-22 levels was found between BCG alone vaccinated and pre-exposure boosted groups. Addition of adjuvant MPL either enhanced or had no effect on cytokines induced in BALs by boosting with LP-ESAT-6 subunit vaccine (Figure 2).

### Boosting with LP-ESAT-6 subunit vaccine enhances the infiltration of T cells in BALs

**Figure 3:**
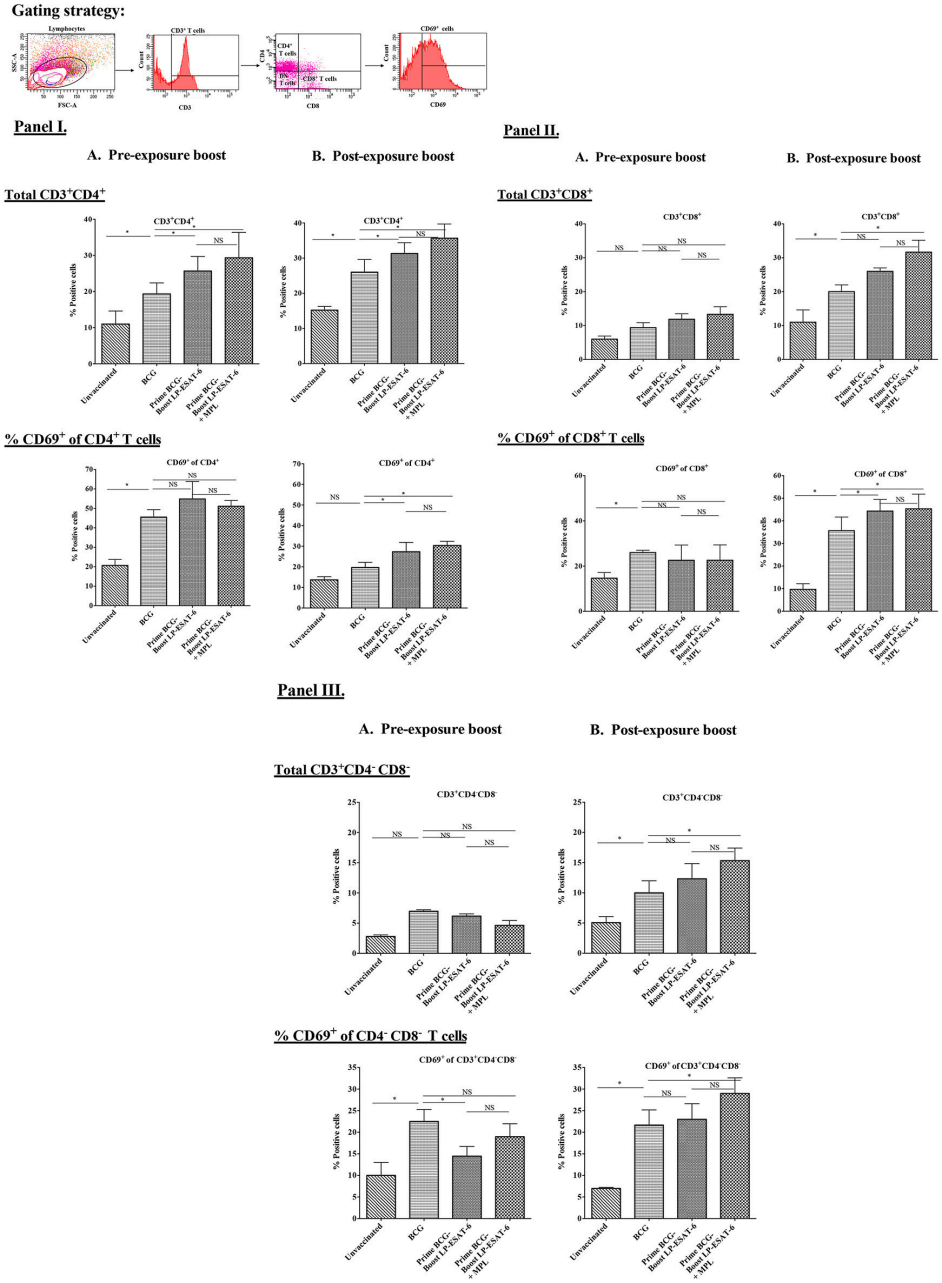
Boosting with LP-ESAT-6 subunit vaccine in BCG-primed mice leads to enhanced recruitment of immune cells in bronchoalveolar lavage fluids. Female BALB/c mice (*n* = 5/group) were immunized according to the pre-exposure or post-exposure schedule shown in Figure 1. Four weeks after infection, mice were euthanized and lung lavages were collected from **(A)** pre-exposure boost and **(B)** post-exposure boost groups to examine the presence and activation (CD69^+^) of CD3^+^CD4^+^ **(I)**, CD3^+^CD8^+^ **(II)** and CD3^+^CD4^−^CD8^−^ (DN) T cells **(III)** by flow cytometry. The gating strategy shown above the bar graphs in **(I)** was used to detect the different subsets of T cell based on the expression of CD3, CD4, CD8 and CD69 markers by flow cytometry analysis. Mean ± standard deviations of percent positive cells from five individual mice are shown. ^*^Indicates significant difference (^*^P < 0.05) between the compared groups and “NS” represents not significant (*P* > 0.05). Data are representative of two different repeated experiments.

T cells infiltrating airways have been shown to be critical for improved protection after mucosal vaccination. Thus, we next sought to determine T cell infiltration in BALs to see how pre- and post-exposure LP-ESAT-6 subunit boost might modify the BCG-primed T cell responses in mouse lungs. Due to the presence of multiple T cell epitopes in both BCG as well as LP-ESAT-6 subunit vaccine, we chose to examine gross changes in total T cell population, instead of peptide epitope-specific T cells. The results obtained demonstrated that the frequency of infiltrating CD4^+^ T cells was higher in LP-ESAT-6 subunit boosted mice compared to BCG-alone groups, regardless of the time of boost (Figures 3IA,B). In contrast, frequencies of CD8^+^ and CD4^−^CD8^−^ T cells (double negative T cells, DN T cells) were significantly enhanced in the post-exposure boost group compared to BCG-alone and pre-exposure boost groups (Figures 3II,IIIA,B). Strikingly, a significantly higher frequency of CD8^+^ T cell subsets that expressed activation marker CD69 (CD8^+^CD69^+^) was found in the post-exposure boost group compared to BCG-alone (Figures 3IIA,B). The frequencies of CD4^+^ and CD4^−^CD8^−^ T cells expressing CD69 were found to be comparable in both LP-ESAT-6 pre-and post-exposure subunit boosts (Figures 3I,IIIA,B). Inclusion of MPL in LP-ESAT-6 subunit vaccine led to an overall increase in the infiltration of T cells in BAL irrespective of the schedule of the boost (Figure 3). Overall, these data suggest that boosting BCG-primed responses with LP-ESAT-6 subunit vaccine results in an enhanced infiltration of activated CD4^+^, CD8^+^, and CD4^−^CD8^−^ T cells in BALs, with selectively increased infiltration of activated CD8^+^ T cells in the post-exposure boost schedule. This could at least partially account for the enhanced protection over BCG-alone.

### Schedule of boost with LP-ESAT-6 subunit vaccine distinguishes the proliferative response of splenocytes upon *ex vivo* stimulation with the respective antigen

**Figure 4:**
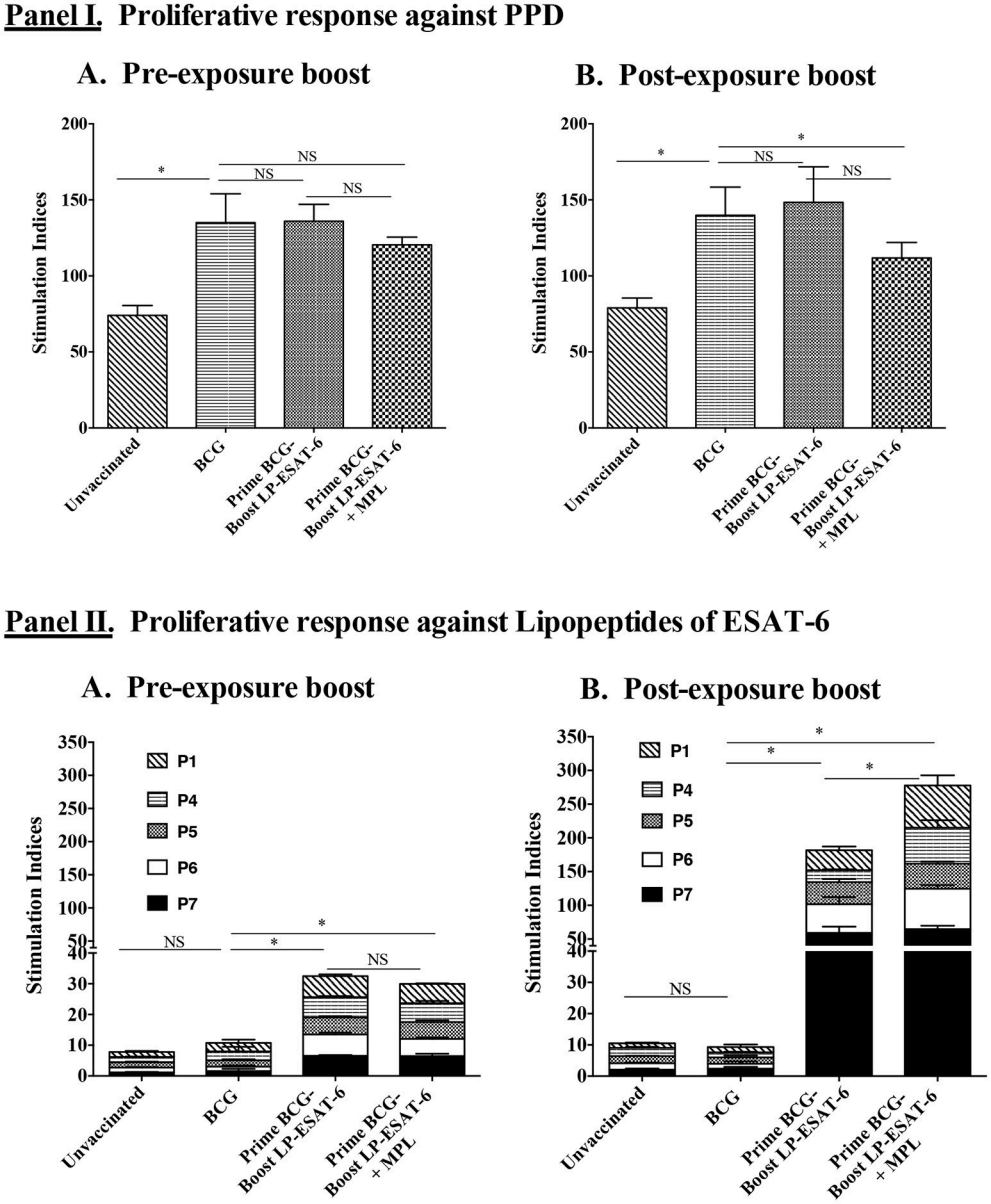
Proliferative responses of splenocytes upon *ex vivo* stimulation with PPD or individual lipopeptide components of LP-ESAT-6 subunit vaccine. Female BALB/c mice (*n* = 5/group) were immunized according to the pre-exposure or post-exposure schedule shown in Figure 1. Four weeks after infection, mice were euthanized and spleens were collected from **(A)** pre-exposure boost and **(B)** post-exposure boost groups to examine the antigen-specific proliferation of T cells. Briefly, T cells obtained from splenocytes (pooled from 5 mice of a group) were cultured with irradiated APCs (splenocytes from unimmunized mice) and with medium, PPD (1 μg/ml) (**Panel I**) or respective lipopeptides P1 and P4-P7 at 10 μg/ml concentration (**Panel II**) for 4 days. T cell proliferation was measured by [^3^H] thymidine incorporation. Stimulation indices were calculated as CPM of the antigen-containing cultures/CPM of medium control group. Mean ± standard deviation of stimulation indices from triplicate wells are shown. ^*^denotes significant difference (*P* < 0.05) between the compared groups and “NS” represents not significant (*P* > 0.05).

Next, we sought to determine whether intranasal boosting with LP-ESAT-6 subunit vaccine at different schedules i.e., pre- and post-exposure, distinguishes the systemic T cell responses induced against the boosting antigen. We performed a bulk T cell proliferation assay using splenocytes obtained from different experimental groups after *ex vivo* stimulation with purified protein derivative (PPD) of *Mtb* or individual lipopeptides of the LP-ESAT-6 subunit vaccine and results are expressed as stimulation indices, obtained by dividing the counts per minute of antigen-stimulated culture with that of the corresponding medium-solvent control culture (Figure 4). Our results demonstrated that the unvaccinated group showed some proliferation in response to PPD antigen due to intrinsic T cell priming upon infection with *Mtb* in the mice. BCG vaccination led to a significant increase in PPD-specific proliferation. Interestingly, however, boosting with LP-ESAT-6 subunit vaccine in the presence or absence of adjuvant MPL did not lead to a significant difference in PPD-specific proliferation, which was also not affected by the schedule of boosting (Figures 4IA,B). In contrast, responses to *ex vivo* stimulation with individual lipopeptide components of the LP-ESAT-6 subunit vaccine were remarkable (Figures 4IIA,B). While there was a significant T cell proliferative response against ESAT-6 lipopeptides in splenocytes from mice which were given a boost before infection compared to the BCG-vaccinated or unvaccinated group, the proliferative response was dramatically increased in mice given the post-exposure boost with LP-ESAT-6 subunit vaccine (Figure 4II). Further, the addition of adjuvant MPL did not result in an enhancement of antigen-specific proliferation in the pre-exposure boost group, but resulted in a stronger enhancement in T cell proliferation in the post-exposure boost group, compared to the respective LP-ESAT-6 subunit group (Figure 4II). These results suggest that immunization with BCG possibly leads to modulation of T cells that compromises response to the subunit vaccine, whereas if the boost is given after *Mtb* infection, it is able to re-educate the immune system and lead to prolific subunit vaccine-induced T cell responses culminating in improved efficacy by reducing the *Mtb* burden.

### Schedule of the boost with LP-ESAT-6 subunit vaccine dictates the quality of systemic CD4^+^ and CD8^+^ T cell responses

**Figure 5:**
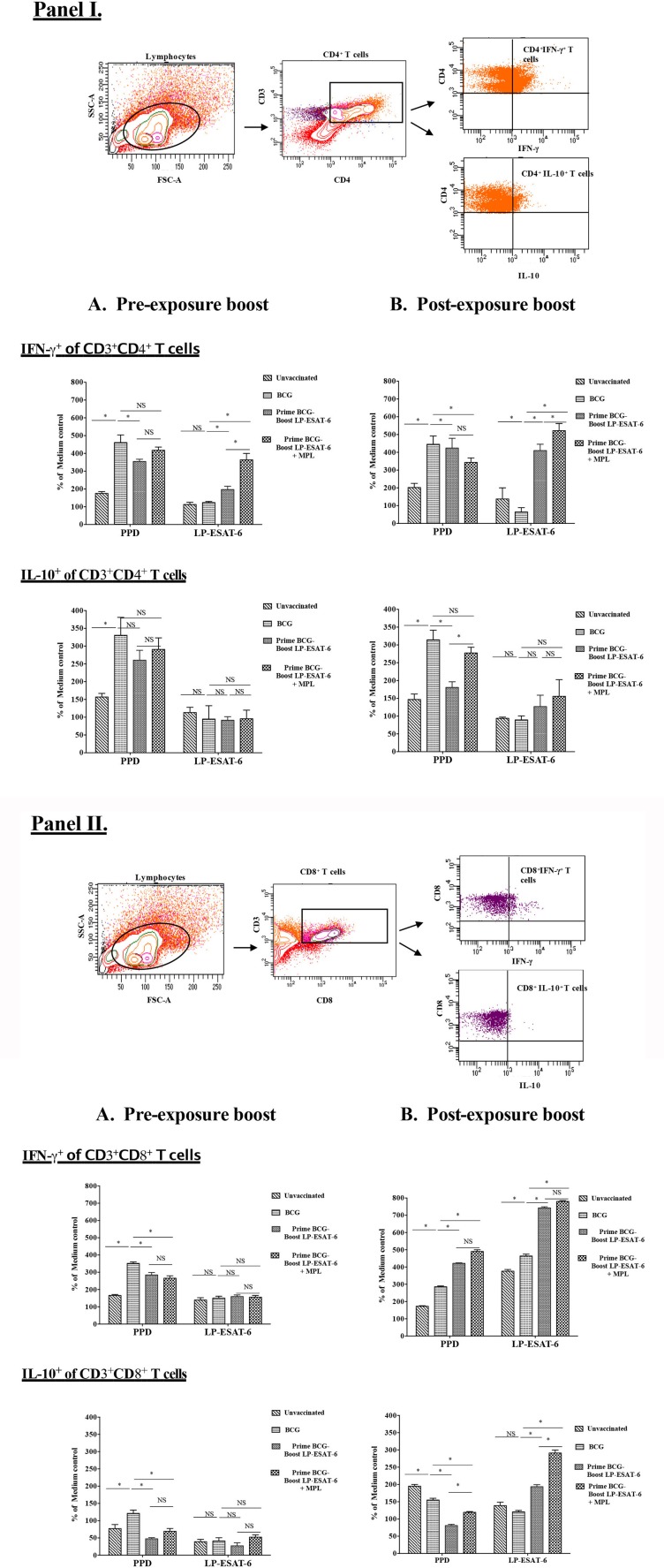
Intracellular IFN-γ and IL-10 are differentially expressed in antigen-specific CD4^+^ **(I)** and CD8^+^ **(II)** T cells in BCG-primed mice boosted with LP-ESAT-6 subunit vaccine in pre-exposure or post-exposure schedule. Female BALB/c mice (*n* = 5/group) were immunized according to the pre-exposure or post-exposure schedule shown in Figure 1. Four weeks after infection, mice were euthanized and spleens were collected from pre-exposure boost and **(B)** post-exposure boost groups. Spleen cells obtained from 5 mice immunized with lipopeptide P1 and P4-P7 were cultured for 4 days with or without PPD (1 μg/ml) or lipopeptide mix (each of P1 and P4-P7 at 1 μg/ml), cultured with Brefeldin A (1.5 μg/ml) 1X; eBioscience) for 5 h, and labeled with antibodies against CD3, CD4 and CD8 for extracellular staining along with intracellular IFN-γ and IL-10. The cells were gated for CD3^+^CD4^+^ and CD3^+^CD8^+^, which were subsequently analyzed for IFN-γ or IL-10 expression as shown in the gating strategy shown above the bar graphs in each panel. Data are shown as the percentage of IFN-γ^+^ or IL-10^+^ of CD4^+^ **(I)** and CD8^+^ T cells **(II)** upon stimulation with PPD or lipopeptide mix normalized to medium control in each of the experimental groups: unvaccinated, BCG alone, BCG prime/LP-ESAT-6 boost and BCG prime/LP-ESAT-6+MPL boost in **(A)** pre-exposure and **(B)** post-exposure schedules. Mean ± standard deviation from triplicate cultures from spleen cells pooled from five individual mice are shown at the bottom. ^*^denotes significant difference (*P* < 0.05) between the compared groups and “NS” represents not significant (*P* > 0.05). Data are representative of two repeated experiments.

The functional quality of T cell responses induced with a subunit vaccine boost would determine their protective efficacy and ability to boost BCG-primed responses. We observed that a post-exposure boost with LP-ESAT-6 subunit vaccine provided higher reduction in *Mtb* viable counts, higher IFN-γ levels in the BALs and higher proliferation of splenocytes in antigen-dependent manner compared to BCG vaccination only and pre-exposure boost (Figures 1, 2, 4). We then sought to examine the functional attributes of CD4^+^ and CD8^+^ T cells in splenocytes stimulated *ex vivo* with PPD or LP mix as recall antigens (Figure 5). Bulk splenocyte cultures incubated with medium, PPD or LP mix for 4 days were used to determine intracellular cytokine production in CD4^+^ and CD8^+^ T cells by flow cytometry. The data are expressed as % of medium control culture in the corresponding experimental groups, to determine the antigen specificity/dependency of the induced cytokine responses. Among the CD4^+^ T cells, IFN-γ induced in response to *ex vivo* stimulation with PPD was increased in the BCG-immunized group compared to the unvaccinated group, but was unaffected in the groups with an LP-ESAT-6 boost both in pre- and post-exposure groups (Figures 5IA,B). In contrast, there was a significant increase in IFN-γ-producing CD4^+^ T cells in the LP-ESAT-6 post-exposure groups, both compared to pre-exposure, unvaccinated and BCG only vaccinated groups (Figures 5IA,B). Interestingly, in PPD recall groups an increase in IL-10-producing CD4^+^ T cells was observed upon BCG vaccination, which was maintained upon boosting with LP-ESAT-6 in both schedule. In contrast, however, *ex vivo* stimulation of splenocytes with LP mix did not lead to a significant increase in IL-10-producing CD4^+^ T cells in unvaccinated, BCG-alone and LP-ESAT-6±MPL groups, suggesting that boosting with the LP-ESAT-6 subunit did not lead to CD4^+^ T cells producing high levels of IL-10 (Figures 5IA,B). Examination of cytokines produced in CD8^+^ T cells demonstrated an entirely different but interesting pattern (Figures 5IIA,B). While in response to PPD there was induction of IFN-γ and IL-10-producing CD8^+^T cells after BCG vaccination, they were similar in LP-ESAT-6-boosted groups irrespective of the schedule of boosting. In contrast, in response to *ex vivo* stimulation with ESAT-6 LP mix, only in the post-exposure group there was a significantly higher % of IFN-γ as well as IL-10 producing CD8^+^ T cells, which were further enhanced in the groups that received MPL in addition to LP-ESAT-6 vaccine (Figures 5IIA,B). There seemed to be almost a complete lack of induction of antigen-specific/dependent IFN-γ and IL-10-producing CD8^+^ T cells in pre-exposure boost groups, which received LP-ESAT-6 subunit vaccine with or without MPL. These data indicate that there is a significant difference in the induction of effector CD8^+^ T cells depending on the timing of the subunit vaccine given pre- or post-exposure. It is not clear whether this difference is due to immunomodulation induced by BCG vaccine, which cannot be overcome by a subunit vaccine as such. But upon infection, the subunit vaccine is either able to amplify the primary response induced by infection or the immunomodulation induced by BCG vaccine is overcome by the productive infection.

### Boosting with LP-ESAT-6 subunit vaccine enhances granzyme B (GrB)-producing effector lymphocytes including NK, NKT and CD8^+^ T cells

**Figure 6:**
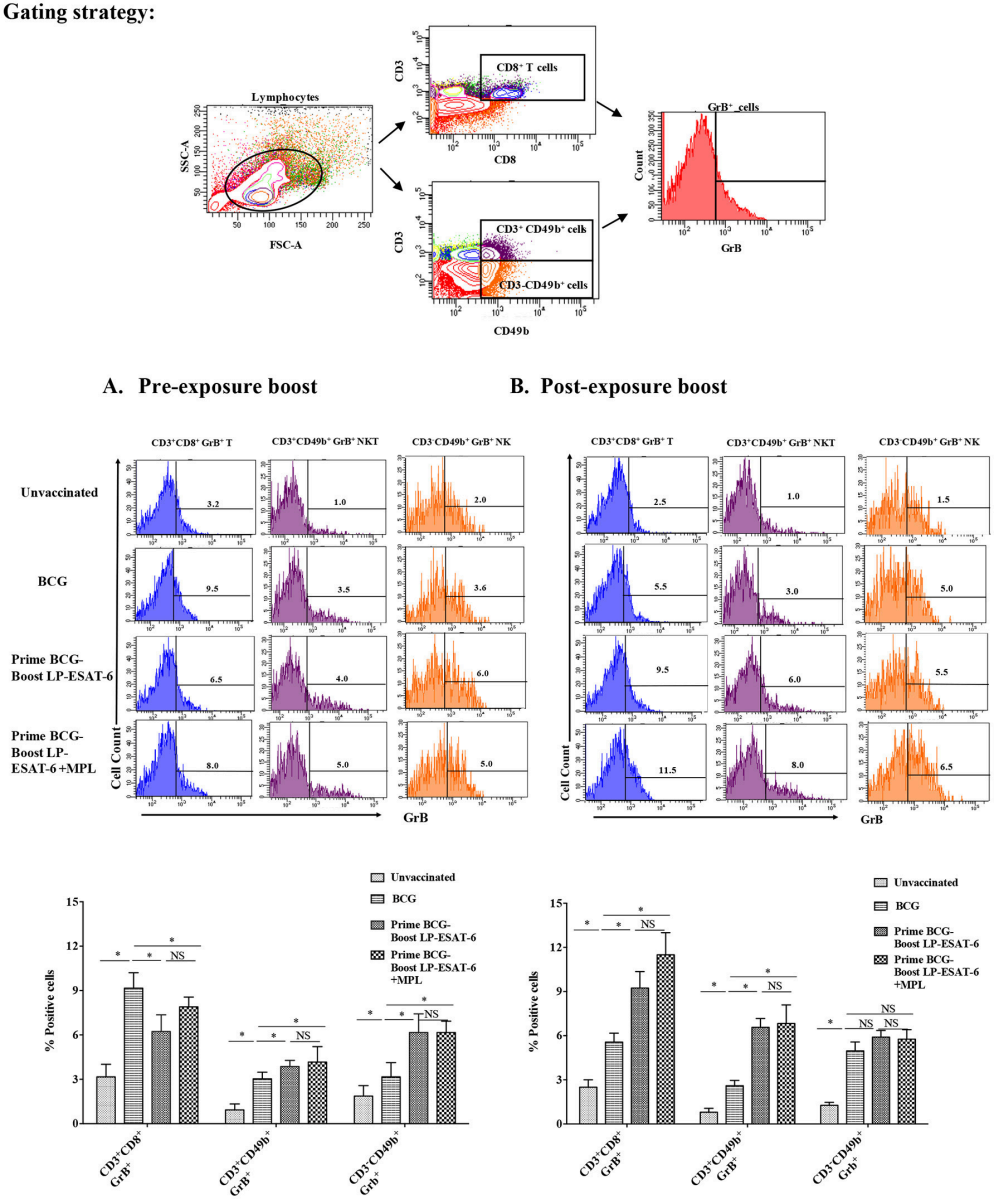
Induction of granzyme B (GrB)-producing effector lymphocytes including NK and NKT and CD8+ T cells in BCG-primed mice boosted with LP-ESAT-6 subunit vaccine in the pre-exposure or post-exposure schedule. Female BALB/c mice (*n* = 5/group) were immunized according to the pre-exposure or post-exposure schedule shown in Figure 1. Four weeks after infection, mice were euthanized and spleens were collected from **(A)** pre-exposure boost and **(B)** post-exposure boost groups. Spleen cells obtained from individual mice were cultured for 5 h with Brefeldin A (1.5 μg/ml) 1X; eBioscience), and labeled with antibodies against CD3, CD8 and CD49b for extracellular staining along with intracellular GrB. The cells were gated for CD3^+^CD8^+^, CD3^−^CDCD49b^+^ (NK) and CD3^+^CDCD49b^+^ (NKT) cells, which were subsequently analyzed for GrB expression as shown in the gating strategy above the bar graphs in each panel. Data are shown as the percentage of GrB^+^ of CD3^+^CD8^+^, CD3^−^CDCD49b^+^ and CD3^+^CDCD49b^+^ cells from each of the experimental groups: unvaccinated, BCG alone, BCG prime/LP-ESAT-6 boost and BCG prime/LP-ESAT-6+MPL boost in **(A)** pre-exposure and **(B)** post-exposure schedules. Histograms of GrB^+^ cells are shown in the top panel and bar graphs representing mean ± standard deviations from five individual mice are shown in the bottom panel in each of the schedules **(A,B)**. ^*^denotes significant difference (*P* < 0.05) between the compared groups and “NS” represents not significant (*P* > 0.05). Data are representative of two repeated experiments.

For an intracellular pathogen like *Mtb*, besides effector cytokines, cytotoxic mechanisms are important to rid the infected host cells of bacteria, and Granzyme B (GrB) is a marker of functional effector cytotoxic cells. NK, NKT and CD8^+^ T cells are prominent cytotoxic lymphocytes and mediate killing of infected cells through the secretion of GrB. NK and NKT cells have been classically considered as innate immune cells but have recently been also shown to be stimulated upon antigen-based vaccinations and have memory responses like adaptive T cells (33). To examine the effect of LP-ESAT-6 subunit vaccine boost on effector cytotoxic lymphocytes, splenocytes obtained from mice were examined for GrB expression in NK, NKT and CD8^+^ T cells without *ex vivo* re-stimulation (Figures 6A,B). Intriguingly, the percent of CD8^+^T cells expressing GrB increased in the order: unvaccinated <BCG alone <LP-ESAT-6 boost <LP-ESAT-6+MPL boost in the post-exposure schedule, whereas in the pre-exposure schedule, boosting with LP-ESAT-6±MPL led to a decrease compared to the BCG group (Figures 6A,B). GrB expression in both NK (CD3^−^CD49b^+^) and NKT cells (CD3^+^CD49b^+^) showed a trend as unvaccinated <BCG alone <LP-ESAT-6 boost <LP-ESAT-6+MPL in both pre- and post-exposure groups (Figures 6A,B). These experiments indicate that boost with LP-ESAT-6 subunit vaccine leads to overall increase in GrB-expressing effector cytotoxic cells compared to BCG alone.

## Discussion

There is clearly an urgent unmet need to investigate new vaccine/immunotherapy approaches for tuberculosis. Heterologous prime-boost vaccination appears to be a promising strategy in the investigation of new vaccine approaches. However, despite an increased focus in the last two decades, clinical progress in heterologous prime-boost strategies has been limited due to insufficient understanding of the protective immune response required, target antigen and its delivery, and route and schedule of boosting. While viral vector derived boosting vaccines have been investigated, there has been limited clinical success and concerns of serious side effects (12–18). A number of fusion protein-based subunit vaccines are being tested as boosters to BCG. However, weak immunogenicity of protein/peptide-based vaccines requires a safe and effective adjuvant, which in itself poses issues (19–24, 34).

Our earlier studies demonstrated that lipid-modified permissive T cell epitopes from ESAT-6 (LP-ESAT-6) induce strong protective immunity against *Mtb* upon mucosal (intranasal) administration, which is further enhanced by addition of an adjuvant MPL (30). With the premise that ESAT-6-based subunit vaccine by itself may not be sufficient to induce broad multifunctional protective immunity required to protect from a complex pathogen such as *Mtb*, we sought to investigate its potential as a boosting or supplementing vaccine after priming with BCG. This approach would not stringently conform to the classical definition of boosting since BCG vaccine does not encompass ESAT-6 antigen, but would rather provide an expansion to the concept of prime-boost vaccine strategies. However, numerous earlier examples of not-so-successful preclinical and clinical testing with BCG-prime and heterologous-boost prompted us to re-examine this approach in an unconventional manner. Most of the post-exposure or therapeutic vaccines previously studied were evaluated in BCG-naïve or BCG-vaccinated healthy individuals (14, 18, 19). Traditional prime-boost vaccine approaches require administration of the booster vaccine at various times after the priming vaccine to prevent an infection. But the timing, dose and route of which still continues to be debated. Although BCG-induced immunity is known to wane after time, it is known to be good for 10–15 years after childhood immunization in humans (6, 35, 36). This suggests that only minor changes in the timing of booster vaccination may not show quantifiable differences. While there are scarce studies, which thoroughly examined the timing of boosting, homologous boosting with BCG after BCG priming has not demonstrated beneficial effect compared to heterologous boosting (37). Effect of timing with different boosting vaccines has been variable: the boosting potential of MVA85A was not found to be influenced by the timing of the boost after BCG-priming in children whereas prolonging the time between BCG priming and boosting was found to be favorable for another subunit vaccine that used a recombinant mycobacterial adhesin heparin-binding haemagglutinin (rHBHA) (38). It has been speculated that repeated activation of already activated T cells may lead to increased apoptosis following a subunit-boost resulting in a suboptimal booster effect observed at the peak of a BCG response, whereas boosting at the contraction/effector memory phase of a T cell response may provide a better effect (39–41). However, for a heterologous boosting antigen (ESAT-6), which is absent in BCG, these mechanisms may neither be relevant nor limiting, and therefore it may offer an advantage due to at least not having a detrimental effect caused by apoptosis of already activated T cells. Further, BCG is known to not protect against pulmonary TB and our LP-ESAT-6 subunit vaccine demonstrated promising efficacy against pulmonary *Mtb* infection when given intranasally. Above all, since many of the BCG-primed individuals still develop active *Mtb* infection and TB disease, and one third of the world's population is latently infected with *Mtb*, there is a desperate need to develop post-exposure vaccine for TB.

Our experimental protocol (Figures 1IA,B), was designed to test LP-ESAT-6 as both a prophylactic (pre-exposure) and a therapeutic (post-exposure) vaccine in mice primed with BCG. Surprisingly, LP-ESAT-6 subunit vaccine was not able to confer enhanced protection in the pre-exposure schedule (except in liver) but exhibited promising enhancement of protection in the post-exposure schedule when compared to the BCG-alone group (Figures 1IIA,B). It is possible that LP-ESAT-6 vaccination post-infection also leads to reduction in *Mtb* loads in BCG unprimed mice, however, this group was not included due to our experimental design focusing on BCG primed animals. The lack of improvement in protection in the pre-exposure schedule was unexpected as in our earlier studies LP-ESAT-6 subunit vaccine by itself conferred significant protection against *Mtb* infection (30) and we were anticipating at least an additive effect. The exact mechanism for this discrepancy is not clear. However, we speculate that priming with BCG modulates and regulates T cells in a way that could not be overcome by LP-ESAT-6 subunit boost prior to infection, whereas upon infection, either T cells get primed due to *de novo* infection with *Mtb* which then get boosted upon administration of the LP-ESAT-6 subunit vaccine or the productive infection allows re-education of recent thymic emigrant (RTE) T cells. These possibilities will be investigated in future studies. It has been shown in a mouse model of *Mtb* infection that recent thymic emigrants contribute to peripheral T cell responses during acute *Mtb* infection (42). On the other hand, chronic infection with *Mtb* leads to exhaustion of antigen-specific T cells. Further, central tolerance is not a major factor limiting T cell responses during *Mtb* infection, and infection with *Mtb* is capable of supporting the activation of recent thymic emigrants (42), opening the possibility of re-educating them with a subunit booster vaccine. Although BCG vaccine is a live vaccine and expected to replicate in the host, the short duration (14 days) between BCG priming and LP-ESAT-6 subunit vaccine in the pre-exposure schedule in our study is not expected to lead to exhaustion or apoptosis of T cells. Furthermore, since ESAT-6 antigen is not present in the BCG vaccine strain, these mechanisms should not blunt the response induced against ESAT-6 antigen. It is possible that priming with BCG induced T_regs_, which then dampened the induction of responses against LP-ESAT-6. It has been shown earlier that BCG vaccine triggers the specific activation of CD4^+^ and CD8^+^ T_regs_ (43–45). However, to what extent the T_regs_ affect boosting with another vaccine is not clear, and we did not evaluate them in our experiments as we performed all of our studies in mice with productive infection with *Mtb*.

The examination of BAL fluid for cytokines demonstrated an interesting pattern in IFN-γ vs. IL-17A production (Figures 2A,B). While IFN-γ has been suggested to be the single most important cytokine in protective immunity against *Mtb*, the role of IL-17A could be protective, inflammatory and/or regulatory (46). Compared to healthy people, CD4^+^ T cells producing IL-22 and IL-17A have been reported to be upregulated in BAL fluids of pulmonary TB patients (47), indicating their probable contribution to anti-mycobacterial responses. Interestingly, the post-exposure boost in our study led to enhanced IFN-γ, IL-17A and IL-22 levels, in contrast to pre-exposure boost, where IL-17A and IL-22 was enhanced in the absence of upregulation in IFN-γ; IL-10 level was reduced proportionally. The exact cellular source of these cytokines in BAL fluids is also not clear and could be from innate and/or adaptive lymphocytes. The examination of BAL infiltrating lymphocytes demonstrated an interesting pattern with a clear distinction between pre-exposure and post-exposure boost (Figures 3I,II,IIIA,B). While BCG priming led to increased recruitment of CD4^+^ T cells compared to unvaccinated mice, boosting with ESAT-6^+^MPL led to the significant enhancement in CD8^+^ and CD4^−^CD8^−^ (double negative, DN) T cells infiltrating BAL only in the post-exposure boost regimen. The reason why there was no enhancement in CD8^+^ and CD4^−^CD8^−^ T cells infiltrating BAL in the pre-exposure boost schedule, is not clear. However, it appears that the increased CD8^+^ and CD4^−^CD8^−^ T cells in BAL in the post-exposure boost may at least be partially contributing to increased reduction in *Mtb* viable counts (Figures 1, 3). For an intracellular pathogen like *Mtb*, besides cytokine-mediated mechanisms, cytotoxic lymphocytes are expected to play a significant role in removing the infected host cells, thereby clearing the infection (48). Interestingly, BCG vaccine has been known to predominantly induce CD4^+^ T cells and attempts to broaden the response toward both CD4^+^ and CD8^+^ T cell responses have included boosting with recombinant adenovirus vector containing Ag85A and B, and making recombinant BCG more amenable to inducing CD8^+^ T cell response by including multiple antigens and perfringolysin (49–52). BCG is also known to induce CD8^+^ T_regs_ (43, 53), which may have compromised the ability of the LP-ESAT-6 boost to enhance CD8^+^ T cell infiltration in the pre-exposure boost, but which could be overcome by productive infection with *Mtb* resulting in a significant increase in the post-exposure boost. Further, in the post-exposure boost with LP-ESAT-6, there was a significant increase in double negative T cells (CD3^+^CD4^−^CD8^−^) in BALs. The expression of early activation marker CD69 on both CD8^+^ and DN T cells, although increased over unvaccinated mice, was not significantly different compared to the BCG-alone group. Since CD69 is expressed transiently early after activation of T cells, the timing of experiments could have contributed to seemingly no significant increase. The significant enhancement in percentage of DN T cells in BAL upon post exposure boost is interesting, although the current studies did not clearly identify the function of these cells in contributing to the reduction of *Mtb* loads. Double negative T cells have been suggested to be of thymic and extra-thymic origin, prevalent in lungs, liver and genital tract, and may have inflammatory and/or regulatory potential (54). Interestingly, in response to a live vaccine against respiratory infection with intracellular bacteria *Francisella tularensis* in mice, DN T cells were shown to be the major responding T cells (55). DN T cells have been shown to produce IL-17A earlier than CD4 T cells and demonstrate features of antigen-experienced T cells including lower threshold of stimulation, proliferation, cytokine production, and also contribute to clearance of infection (56). Further, IL-10 has been shown to limit the expansion of DN T cells (57). Our results also demonstrated a reciprocal regulation of IL-10 and DN T cells in BALs in post-exposure boosted mice (Figures 2, 3). Double negative memory T cells in lungs with functional properties similar to CD8^+^ T cells have also been shown to react to influenza virus infection. Further, they were shown to express CD69, representing the activated memory phenotype (58). Overall, our results demonstrated an enhancement of CD8^+^ and DN T cells in BAL upon post-exposure boosting with LP-ESAT-6±MPL, which may have contributed to increased reduction in *Mtb* loads.

Proliferation and expansion of antigen-specific T cells is a signature of the activation of adaptive immunity. In gross splenocyte proliferation assays, the recall response to purified protein derivative (PPD) did not show an enhancement in the LP-ESAT-6-boosted group when compared to BCG alone. However, recall response to individual LPs of the LP-ESAT-6 subunit vaccine was substantially increased in the post-exposure group, compared to pre-exposure group (Figure 4). Addition of MPL further enhanced this effect (Figure 4). Our results demonstrated that the T cell proliferation response to ESAT-6 was very poor in the pre-exposure group where LP-ESAT-6 was given 14 days after BCG priming (Figure 4II). This result was in contrast to our earlier studies where LP-ESAT-6±MPL immunized mice, which were not primed with BCG, showed excellent T cell responses against LP-ESAT-6 (30); this was likely due to immune-modulation induced by priming with BCG vaccine. We observed that proliferation in response to PPD recall was not affected in both pre- and post-exposure boost with the subunit compared to the BCG-alone group. But the proliferative response to LPs became very highly exaggerated in the post-exposure boost, further indicating that LP-ESAT-6 subunit vaccine was not efficient in boosting the BCG priming *per se* but either could re-educate the *Mtb*-specific response after infection or that the productive infection could overcome the BCG-induced regulatory effects.

Analyses of antigen-dependent IFN-γ and IL-10 production in CD4^+^ and CD8^+^ T cells from *ex vivo*-stimulated splenocytes of BCG-alone and LP-ESAT-6 subunit vaccine boost groups (Figures 5I,IIA,B) demonstrated clear superiority of post-exposure LP-ESAT-6 subunit vaccine in inducing IFN-γ producing CD8^+^ T cells, compared to pre-exposure boost. IFN-γ-producing CD8^+^ T cells have been demonstrated to be efficient effectors for intracellular pathogens (59). Intriguingly though, there also was a significant increase in IL-10-producing CD8^+^ T cells (Figure 5II). In a mouse model of coronavirus infection-induced encephalitis, IL-10 produced by CD8^+^ T cells has been demonstrated to reflect cytotoxic T lymphocytes with a superior effector (cytolytic) function (60). Unlike IL-10 production by CD4^+^ T cells, which have been shown to be at least partially associated with persistent infections, IL-10 producing CD8^+^ T cells have been shown in acute respiratory infections with influenza virus, respiratory syncytial virus and simian virus (61–63).

Granzyme B, produced by activated innate and adaptive cytotoxic lymphocytes, is a determinant of cytotoxic granules and perforin-dependent cytolytic activity. Clearance of an intracellular pathogen such as *Mtb* would be highly facilitated by activation of cytotoxic lymphocytes. We conducted GrB expression analyses in splenocytes directly obtained from the animals without *ex vivo* culturing, so as to avoid non-specific *ex vivo* activation of effector lymphocytes. It has been reported that cytokines such as IL-2 and IL-15 can stimulate GrB expression in T cells even without antigen stimulation (64). Our examination of intracellular GrB expression in splenocytes demonstrated that boosting with LP-ESAT-6 subunit vaccine increased GrB expression in CD8^+^ T cells compared to BCG vaccination alone in the post-exposure boost group but not in the pre-exposure group (Figure 6). In contrast, GrB expression on NK (CD3-CD49b^+^) and NKT (CD3^+^CD49b^+^) cells was upregulated following BCG vaccination compared to the unvaccinated group, which showed a trend toward further enhancement with LP-ESAT-6 boosting in both pre and post-exposure schedules (Figure 6). These results indicate that boosting with LP-ESAT-6 may be inducing memory-like effects in NK and NKT cells. It has been reported that homologous BCG boosting in adult humans, besides stimulating CD4^+^ and CD8^+^ T cells, also stimulates memory NK and NKT cells expressing GrB as an antimycobacterial effector molecule (33).

A key question in attempts to generate a more effective TB vaccine is the quality and quantity of immune responses required for optimal protection against TB. Our studies clearly suggest that mucosal boosting with LP-ESAT-6 subunit vaccine allows the expansion of immune responses with respect to the target mycobacterial antigen, and overall quality and quantity, compared to BCG vaccine alone. A less-explored but crucial question is the timing of the boost after BCG priming. Our studies have shed important light on this key question and perhaps partially help to explain the many unsuccessful attempts directed toward a prime-boost strategy for TB vaccine. Further work is needed to determine the detailed reasons/mechanisms for the inability of a pre-exposure boost with LP-ESAT-6 subunit vaccine to enhance protection compared to BCG alone. Nevertheless, they represent a significant advancement demonstrating that scheduling the subunit vaccine boost post-exposure, and therefore re-educating the T cells, may be a promising future path toward a successful TB vaccine. Additionally, these studies provide immense promise for development of a subunit vaccine for BCG-vaccinated individuals who still get active TB infection.

## Author contributions

RK, NG, DK, and BA: Conception; NG, SG, SV, and RK: Experimental planning and execution; NG, RK, and BA: Data analyses and manuscript writing.

### Conflict of interest statement

The authors declare that the research was conducted in the absence of any commercial or financial relationships that could be construed as a potential conflict of interest.

## References

[1] WHO Global tuberculosis report 2017. (2017). Available online at: http://www.who.int/tb/publications/global_report/en/

[2] Dorman S Chaisson R. From magic bullets back to the magic mountain: the rise of extensively drug-resistant tuberculosis. Nat Med. (2007) 13:295–98. 10.1038/nm0307-295 17342143

[3] Dye C Scheele S Dolin P Pathania V Raviglione M. Consensus statement. Global burden of tuberculosis: estimated incidence, prevalence, and mortality by country. WHO Global Surveillance and Monitoring Project. JAMA (1999) 282:677–86. 10.1001/jama.282.7.677 10517722

[4] Fine PE. Variation in protection by BCG: implications of and for heterologous immunity. Lancet (1995) 346:1339–45. 10.1016/S0140-6736(95)92348-9 7475776

[5] Young B Perkins M Duncan K Barry CE. Confronting the scientific obstacles to global control of tuberculosis. J Clin Invest. (2008) 118:1255–65. 10.1172/JCI34614 18382738 PMC2276805

[6] Mangtani P Abubakar I Arti C Beynon R Pimpin L Fine PE Protection by BCG vaccine against tuberculosis: a systemic review of randomized controlled trials. Clin Infect Dis. (2014) 58:470–80. 10.1093/cid/cit790 24336911

[7] Trunz B Fine P Dye C. Effect of BCG vaccination on childhood tuberculous meningitis and miliary tuberculosis worldwide: a meta-analysis and assessment of cost-effectiveness. Lancet (2006) 367:1173–80. 10.1016/S0140-6736(06)68507-3 16616560

[8] Andersen P Doherty TM. The success and failure of BCG-implications for a novel tuberculosis vaccine. Nat Rev Microbiol. (2005) 3:656–62. 10.1038/nrmicro1211 16012514

[9] Andersen P Woodworth JS. Tuberculosis vaccines–rethinking the current paradigm. Trends Immunol. (2014) 35:387–95. 10.1016/j.it.2014.04.006 24875637

[10] Costa AC Nogueira V Kipnis A Junqueira-Kipnis AP. Recombinant BCG: innovations on an old vaccine. scope of BCG strains and strategies to improve long-lasting memory. Front Immunol. (2014) 5:152. 10.3389/fimmu.2014.00152 24778634 PMC3984997

[11] Hoft DF Blazevic A Selimovic A Turan A Tennant J Abate G. Safety and immunogenicity of the recombinant BCG vaccine AERAS-422 in healthy BCG-naïve adults: a randomized, active-controlled, first-in-human Phase 1 trial. EBioMedicine (2016) 7:278–86. 10.1016/j.ebiom.2016.04.010 27322481 PMC4909487

[12] Brennan M Clagett B Fitzgerald H Chen V Williams A Izzo A. Preclinical evidence for implementing a prime-boost vaccine strategy for tuberculosis. Vaccine (2012) 30:2811–23. 10.1016/j.vaccine.2012.02.036 22387630 PMC3335191

[13] Smaill F Xing Z. Human type 5 adenovirus-based tuberculosis vaccine: is the respiratory route of delivery the future? Expert Rev Vaccines (2014) 13:927–30. 10.1586/14760584.2014.929947 24935214

[14] Sheehan S Harris SA Satti I Hokey D Stocdale L Minhinnick A. A phase I, open-label trial, evaluating the safety and immunogenicity of candidate tuberculosis vaccines AERAS-402 and MVA85A, administered by prime-boost regime in BCG-vaccinated healthy adults. PLoS ONE (2015) 10:e0141687. 10.1371/journal.pone.0141687 26529238 PMC4631471

[15] Stylianou E Griffiths K Poyntz H Kandt R Dicks M Stockdale L. Improvement of BCG protective efficacy with a novel chimpanzee adenovirus and a modified vaccinia Ankara virus both expressing Ag85A. Vaccine (2015) 33:6800–08. 10.1016/j.vaccine.2015.10.017 26478198 PMC4678294

[16] Roediger K Kugathasan K Zhang X Lichty B Xing Z. Heterologous boosting of recombinant adenoviral prime immunization with a novel vesicular stomatitis virus-vectored tuberculosis vaccine. Mol Ther. (2008) 16:1161–69. 10.1038/mt.2008.59 18388911 PMC7185538

[17] Zhang M Dong C Xiong S. Vesicular stomatitis virus-vectored multi-antigen tuberculosis vaccine limits bacterial proliferation in mice following a single intranasal dose. Front Cell Infect Microbiol. (2017) 7:34. 10.3389/fcimb.2017.00034 28224119 PMC5293745

[18] Tameris M Hatherill M Landry BS Scriba J Snowden M Lockhart S. Safety and efficacy of MVA85A, a new tuberculosis vaccine, in infants previously vaccinated with BCG: a randomised, placebo-controlled phase 2b trial. Lancet (2013) 381:1021–28. 10.1016/S0140-6736(13)60177-4 23391465 PMC5424647

[19] McShane H. Tuberculosis vaccines: beyond bacille Calmette–Guérin. Philos Trans R Soc Lond B Biol Sci. (2011) 366:2782–89. 10.1098/rstb.2011.0097 21893541 PMC3146779

[20] Luabeya K Kagina M Tameris D Geldenhuys H Hoff T Shi Z. First-in-human trial of the post-exposure tuberculosis vaccine H56:IC31 in *Mycobacterium tuberculosis* infected and non-infected healthy adults. Vaccine (2015) 33:4130–40. 10.1016/j.vaccine.2015.06.051 26095509

[21] Skeiky YA Dietrich J Lasco TM Stagliano K Goetz MA Cantarero L. Non-clinical efficacy and safety of HyVac4:IC31vaccine administered in a BCG prime–boost regimen. Vaccine (2010) 28:1084–93. 10.1016/j.vaccine.2009.10.114 19896449

[22] Norrby M Vesikari T Lindqvist L Maeurer M Ahmed R Mahdavifar S. Safety and immunogenicity of the novel H4:IC31 tuberculosis vaccine candidate in BCG-vaccinated adults: two phase I dose escalation trials. Vaccine (2017) 35:1652–61. 10.1016/j.vaccine.2017.01.055 28216183

[23] Leroux R Forgus S Boever F Clement F Demoitie MA Mettens P Improved CD4? T cell responses to *Mycobacterium tuberculosis* in PPD-negative adults by M72/AS01 as compared to the M72/AS02 and Mtb72F/AS02 tuberculosis candidate vaccine formulations: a randomized trial. Vaccine (2013) 31:2196–206. 10.1016/j.vaccine.2012.05.035 22643213

[24] Leroux R Leroux G Ofori O Moris P Clement F Dubois M Evaluation of the safety and immunogenicity of two antigen concentrations of the Mtb72F/AS02(A) candidate tuberculosis vaccine in purified protein derivative-negative adults. Clin Vaccine Immunol. (2010) 17:1763–71. 10.1128/CVI.00133-10 20861328 PMC2976103

[25] Ravan P Demissie A Equale T Wondwosson H Lein D Amoudy A Human T cell responses to the ESAT-6 antigen from *Mycobacterium tuberculosis*. J Infect Dis. (1999) 179:637–45. 10.1086/314640 9952370

[26] Arlehamn S Sidney J Henderson R Greenbaum A James A Moutafsi M. Dissecting mechanisms of immunodominance to the common tuberculosis antigens ESAT-6, CFP10, Rv2031c (hspX), Rv2654c (TB7.7), and Rv1038c (EsxJ). J Immunol. (2012) 188:5020–31. 10.4049/jimmunol.1103556 22504645 PMC3345088

[27] Harboe M Oettinger T Wiker G Rosenkrands I Andersen P. Evidence for occurrence of the ESAT-6 protein in *Mycobacterium tuberculosis* and virulent *Mycobacterium bovis* and for its absence in *Mycobacterium bovis* BCG. Infect Immun. (1996) 64:16–22. 8557334 10.1128/iai.64.1.16-22.1996 PMC173721

[28] Brandt L Elhay M Rosenkrands I Lindblad B Andersen P. ESAT-6 subunit vaccination against *Mycobacterium tuberculosis*. Infect Immun. (2000) 68:791–5. 10.1128/IAI.68.2.791-795.2000 10639447 PMC97206

[29] Langermans A Doherty M Vervenne A Vander T Lyashchenko K Greenwald R Protection of macaques against *Mycobacterium tuberculosis* infection by a subunit vaccine based on a fusion protein of antigen 85B and ESAT-6. Vaccine (2005) 15:2740–50. 10.1016/j.vaccine.2004.11.051 15780721

[30] Gupta N Vedi S Kunimoto DY Agrawal B Kumar R. Novel lipopeptides of ESAT-6 induce strong protective immunity against *Mycobacterium tuberculosis*: routes of immunization and TLR agonists critically impact vaccine's efficacy. Vaccine (2016) 34:5677–88. 10.1016/j.vaccine.2016.08.075 27693020

[31] Krishnadas D Ahn J Han J Kumar R Agrawal B. Immunomodulation by hepatitis C virus-derived proteins: targeting human dendritic cells by multiple mechanisms. Int Immunol. (2010) 22:491–502. 10.1093/intimm/dxq033 20410260

[32] Yu R Koren G Hotten F Kan M. A protocol for the comprehensive flow cytometric analysis of immune cells in normal and inflamed murine non-lymphoid tissues. PLoS ONE (2016) 11:e0150606. 10.1371/journal.pone.0150606 26938654 PMC4777539

[33] Suliman S Geldenhuys H Johnson JL BCG re-vaccination of adults with latent *Mycobacterium tuberculosis* infection induces long-lived BCG-reactive natural killer cell responses. J Immunol. (2016) 4:1100–10. 10.4049/jimmunol.1501996 PMC4976036 27412415

[34] Skwarczynski M Toth I. Peptide-based synthetic vaccines. Chem Sci. (2016) 7:842–54. 10.1039/C5SC03892H 28791117 PMC5529997

[35] Roy A Eisenhut M Harris RJ Rodrigues LC Sridhar S Habermann S. Effect of BCG vaccination against *Mycobacterium tuberculosis* infection in children: systematic review and meta-analysis. BMJ (2014) 349:g4643. 10.1136/bmj.g4643 25097193 PMC4122754

[36] Colditz GA Brewer TF Berkey CS Wilson ME Burdick E Fineberg HV. Efficacy of BCG vaccine in the prevention of tuberculosis. Meta-analysis of the published literature. JAMA (1994) 271:698–702. 10.1001/jama.1994.03510330076038 8309034

[37] Griffin F Mackintosh G Rodgers R. Factors influencing the protective efficacy of a BCG homologous prime-boost vaccination regime against tuberculosis. Vaccine (2006) 24:835–45. 10.1016/j.vaccine.2005.07.033 16098638

[38] Guerrero G Locht C. Recombinant HBHA boosting effect on BCG induced immunity against *Mycobacterium tuberculosis* infection. Clin Dev Immunol. (2011) 2011:730702. 10.1155/2011/730702 21647410 PMC3102518

[39] Soares AP Kwong CK Choice T Hughes J Jacobs G Smit E. Longitudinal changes in CD4+ T-cell memory responses induced by BCG vaccination of newborns. J Infect Dis. (2013) 207:1084–94. 10.1093/infdis/jis941 23293360 PMC3583271

[40] Jelley-Gibbs DM Dibble JP Fillpson S Haynes L Kemp RA Swain SL Repeated stimulation of CD4 e ector T cells can limit their protective function. J Exp Med. (2005) 201:1101–12. 10.1084/jem.20041852 15795235 PMC2213138

[41] Fraser A Schenkel M Jameson S Vezys V Masopust D. Preexisting high frequencies of memory CD8^+^ T cells favor rapid memory differentiation and preservation of proliferative potential upon boosting. Immunity (2013) 39:171–83. 10.1016/j.immuni.2013.07.003 23890070 PMC3979587

[42] Reiley W Wittmer S Ryan L Eaton S Haynes L Winslow G. Maintenance of peripheral T cell responses during *Mycobacterium tuberculosis* infection. J Immunol. (2012) 189:4451–8. 10.4049/jimmunol.1201153 23028057 PMC3819137

[43] Boer C Meijgaarden E Joosten A Ottenhoff M CD8+ regulatory T cells, and not CD4+ T cells, dominate suppressive phenotype and function after *in vitro* live BCG activation of human cells. PLoS ONE (2014) 9:e94192 10.1371/journal.pone.0094192 24714620 PMC3979753

[44] Akkoc T Aydogan M Yildiz A Karakoc E Eifan A Keles S. Neonatal BCG vaccination induces IL-10 production by CD4^+^ CD25^+^ T cells. Pediatr Allergy Immunol. (2010) 21:1059–63. 10.1111/j.1399-3038.2010.01051.x 20977501

[45] Li L Qiao D Zhang X Liu Z Wu C. The immune responses of central and effector memory BCG-specific CD4^+^ T cells in BCG-vaccinated PPD+ donors were modulated by Treg cells. Immunobiology (2011) 216:477–84. 10.1016/j.imbio.2010.09.003 20950889

[46] Torrado E Cooper AM. IL-17 and Th17 cells in tuberculosis. Cytokine Growth Factor Rev. (2010) 21:455–62. 10.1016/j.cytogfr.2010.10.004 21075039 PMC3032416

[47] Scriba T Kalsdorf B Abrahams D Hofmeister J Isaacs F Black G. Distinct, specific IL-17 and IL-22-producing CD4^+^ T cell subsets contribute to the human anti-mycobacterial immune response. J Immunol. (2008) 180:1962–70. 10.4049/jimmunol.180.3.1962 18209095 PMC2219462

[48] Lin PL Flynn JL. CD8 T cells and *Mycobacterium tuberculosis* infection. Sem Immunopathol. (2015) 37:239–49. 10.1007/s00281-015-0490-8 25917388 PMC4439333

[49] Farinacci M Weber S Kaufmann SH. The recombinant tuberculosis vaccine rBCG ΔureC::hly(+) induces apoptotic vesicles for improved priming of CD4^+^ and CD8^+^ T cells. Vaccine (2012) 30:7608–14. 10.1016/j.vaccine.2012.10.031 23088886

[50] Schaible E Winau F Sieling A Fischer K Collins L Hagens K. Apoptosis facilitates antigen presentation to T lymphocytes through MHC-I and CD1 in tuberculosis. Nat Med. (2003) 9:1039–46. 10.1038/nm906 12872166

[51] Grode L Ganoza CA Brohm C Weiner J Eisele B Kaufmann SH Safety and immunogenicity of the recombinant BCG vaccine VPM1002 in phase 1 open-label randomized clinical trial. Vaccine (2013) 18:1340–810. 10.1016/j.vaccine.2012.12.053 23290835

[52] Sun R Skeiky A Izzo A Dheenadhayalan V Imam Z Penn E Novel recombinant BCG expressing perfringolysin O and the over expression of key immunodominant antigens; pre-clinical characterization, safety and protection against challenge with Mtb. Vaccine (2009) 27:4412–2310. 10.1016/j.vaccine.2009.05.048 19500523

[53] Boer MC van Meijgaarden KE Bastid J Ottenhoff TH Joosten SA. CD39 is involved in mediating suppression by *Mycobacterium bovis* BCG-activated human CD8^+^ CD39^+^ regulatory T cells. Eur J Immunol. (2013) 43:1925–32. 10.1002/eji.201243286 23606272

[54] D'Acquisto F Crompton T. CD3+CD4-CD8- (double negative) T cells: saviours or villains of the immune response? Biochem Pharmacol. (2011) 82:333–40. 10.1016/j.bcp.2011.05.019 21640713

[55] Cowley SC Meierovics AI Frelinger JA Iwakura Y Elkins KL. Lung CD4-CD8- double-negative T cells are prominent producers of IL-17A and IFN-gamma during primary respiratory murine infection with *Francisella tularensis* live vaccine strain. J Immunol. (2010) 184:5791–801. 10.4049/jimmunol.1000362 20393138

[56] Kappes DJ He X He X. CD4-CD8 lineage commitment: an inside view. Nat Immunol. (2005) 6:761–6. 10.1038/ni1230 16034433

[57] Hillhouse E Beauchamp C Chabot-Roy G Dugas V Lesage S. Interleukin-10 limits the expansion of immunoregulatory CD4-CD8- T cells in autoimmune-prone non-obese diabetic mice. Immunol Cell Biol. (2010) 88:771–80. 10.1038/icb.2010.84 20603635

[58] Neyt K GeurtsvanKessel CH Lambrecht BN. Double-negative T resident memory cells of the lung react to influenza virus infection via CD11c(hi) dendritic cells. Mucosal Immunol. (2016) 9:999–1014. 10.1038/mi.2015.91 26376363

[59] Lazarevic V Flynn J. CD8^+^ T cells in tuberculosis. Am J Respir Crit Care Med. (2002) 166:1116–21. 10.1164/rccm.2204027 12379557

[60] Trandem K Zhao J Fleming E Perlman S. Highly activated cytotoxic CD8 T cells express protective IL-10 at the peak of *Coronavirus*-induced encephalitis. J Immunol. (2011) 186:3642–52. 10.4049/jimmunol.1003292 21317392 PMC3063297

[61] Palmer M Holbrook C Arimilli S Parks D Alexander-Miller A. IFNγ-producing, virus-specific CD8^+^ effector cells acquire the ability to produce IL-10 as a result of entry into the infected lung environment. Virology (2010) 404:225–30. 10.1016/j.virol.2010.05.004 20627346 PMC2906694

[62] Spender LC Hussell T Openshaw PJ. Abundant IFN-gamma production by local T cells in respiratory syncytial virus-induced eosinophilic lung disease. J Gen Virol. (1998) 79:1751–8. 10.1099/0022-1317-79-7-1751 9680139

[63] Sun J Madan R Karp CL Braciale TJ. Effector T cells control lung inflammation during acute influenza virus infection by producing IL-10. Nat Med. (2009) 15:277–84. 10.1038/nm.1929 19234462 PMC2693210

[64] Tamang DL Redelman D Alves BN Vollger L Bethley C Hudig D. Induction of granzyme B and T cell cytotoxic capacity by IL-2 or IL-15 without antigens: multiclonal responses that are extremely lytic if triggered and short-lived after cytokine withdrawal. Cytokine (2006) 36:148–59. 10.1016/j.cyto.2006.11.008 17188506 PMC1850105

